# Hypertriglyceridemia and Its Association with HbA1c Test: A Prospective *In Vivo* Controlled Study

**DOI:** 10.1155/2019/4784313

**Published:** 2019-02-12

**Authors:** Rene Rodriguez-Gutierrez, Leonardo G. Mancillas-Adame, Giselle Rodríguez-Tamez, Alejandro Diaz Gonzalez-Colmenero, Ricardo Cesar Solis-Pacheco, Ana Sofia Elizondo-Plazas, Karla M. Santos-Santillana, Linda Gonzalez-Sariñana, Victoria Gonzalez-Nava, José Gerardo Gonzalez-Gonzalez

**Affiliations:** ^1^University Hospital “Dr. Jose E. Gonzalez, ” Universidad Autonoma de Nuevo Leon, Endocrinology Division, Department of Internal Medicine, Monterrey 64460, Mexico; ^2^Universidad Autonoma de Nuevo Leon, Plataforma INVEST Medicina UANL-KER Unit Mayo Clinic (KER Unit Mexico), Monterrey 64460, Mexico; ^3^Division of Endocrinology, Diabetes, Metabolism and Nutrition, Department of Medicine, Mayo Clinic, Rochester, MN 55905, USA; ^4^University Hospital “Dr. Jose E. Gonzalez, ” Universidad Autonoma de Nuevo Leon, Research Unit, Monterrey 64460, Mexico

## Abstract

**Background:**

Hypertriglyceridemia and hyperglycemia coexist in 30-60% of patients with diabetes. The impact of hypertriglyceridemia regarding HbA1c assay reliability remains uncertain. Therefore, we conducted a prospective *in vivo* controlled study with the aim of defining the association between triglyceride levels and HbA1c.

**Methods:**

A total of 44 patients with an index-hospital admission diagnosis of diabetic ketoacidosis or hypertriglyceridemia-induced pancreatitis, as a model for acute elevation of triglycerides, were recruited. Blood samples were drawn for the measurement of HbA1c, triglycerides, glucose, and hemoglobin at baseline and subsequently 24 and 48 hours after admission. HbA1c analysis was performed with high-performance liquid chromatography Bio-Rad D10 (NGSP approved).

**Results:**

All patients completed the study protocol. A difference between mean triglycerides from day 0 (baseline) to day 2 of 1567.2 mg/dL was observed. We found a difference between mean serum HbA1c from days 0 to 2 of 0.09% [1 mmol/mol] (*p* = 0.004). Moreover, a weak correlation between the mean difference of HbA1c and triglycerides from baseline to day 2 was found to be statistically significant (*r* = 0.256, *p* = 0.015). None of these findings, however, are clinically significant.

**Conclusion:**

Triglycerides do not impair the interpretation of HbA1c assay. Patients and clinicians can now be confident that hypertriglyceridemia is not an important factor when interpreting HbA1c results.

## 1. Background

Glycated hemoglobin A1c (HbA1c) has been accepted as an accurate and reliable test both to establish diagnosis and to evaluate glycemic control in patients with diabetes [1, 2]. Its value reflects the mean glucose concentration of the preceding 8 to 12 weeks, and when compared to other diabetes diagnostic tests, HbA1c has a less preanalytic and biological variability and is convenient for patients and clinicians as no fasting is required [1, 3]. Consequently, HbA1c has been universally endorsed by clinical practice guidelines and societies as the reference standard to determine if the patient is at or out of the glycemic goal and at the same time to guide treatment decisions regarding changes in the treatment regimen (i.e., intensifying or deintensifying glucose-lowering medications) [4–6].

Ion-exchange high-performance liquid chromatography (HPLC) is considered one of the main certified methods by the National Glycohemoglobin Standardization Program (NGSP) for HbA1c determination. Its reliability, however, is impaired in certain clinical scenarios characterized by an increase or decrease in red blood cell lifespan such as hemolysis, transfusions, high doses of vitamins C and E, dialysis, HIV infection and pregnancy for the latter (i.e., decreased HbA1c levels) and splenectomy, aplastic anemia, increasing age, and iron deficiency for the former (i.e., increased HbA1c levels) [1, 7–9]. Whether other factors may alter HbA1c reliability is uncertain, including but not limited to uremia, salicylates, alcohol, triglycerides, and opiate use. Not recognizing these factors in clinical practice might impact the decision-making process and potentially threaten the patients' health by increasing the burden of treatment or the risk of adverse events (e.g., hypoglycemia) or by inappropriately deintensifying the treatment regimen.

Hypertriglyceridemia is common in patients with diabetes (>30-60%) [10–12], and it is known to be transiently elevated by uncontrolled hyperglycemia usually in the setting of recent diabetes diagnosis or poor glycemic control (due to inadequate insulin activity and lipolysis). The impact of hypertriglyceridemia regarding HbA1c reliability remains uncertain as most evidence stems from *in vitro* studies or individual cases that report discordant results [8, 9]. In addition, triglyceride levels in these reports (typically >1000 mg/dL) do not represent the concentrations commonly observed in patients with diabetes (150-500 mg/dL) [11–13]; in addition, HbA1c was not measured using the accepted standard, HPLC [8, 9]. Still, clinical decisions in the frequent scenario of concurrent hyperglycemia and hypertriglyceridemia have to be made and evaluated in a real-life study; if there is an association between triglyceride levels and HbA1c, it would have important diagnostic and therapeutic implications.

Accordingly, we decided to perform a prospective controlled study to evaluate the possible impact of hypertriglyceridemia on HbA1c estimation. Secondary outcomes were to compare the effect of a fast triglyceride reduction in HbA1c results in diverse ranges of triglyceride levels and in patients with two different types of index-hospital admission diagnoses.

## 2. Methods

### 2.1. Study Participants

From August 2016 to February 2018, hospitalized adult patients (≥18 years) with an index-hospital admission diagnosis of diabetic ketoacidosis or hypertriglyceridemia-induced pancreatitis [14, 15] were considered for inclusion in the study. In addition, participants had to meet the following criteria: (i) triglyceride levels ≥150 mg/dL at admission, (ii) diabetic ketoacidosis treatment protocol following the American Diabetes Association (ADA) recommendations [16], and (iii) hypertriglyceridemia-induced pancreatitis treatment according to the American College of Gastroenterology (ACG) guidelines [14]. Individuals with any of the following entities that impair quantitative or qualitative hemoglobin (Hb), and consequently HbA1c, were excluded: current or previous (within 6 months) anemia (Hb under 13 g/dL in men and 12 g/dL in women) [17], splenectomy, red blood cell transfusion in the previous 6 months, high-dose salicylate intake, pregnancy, HIV infection, hyperuricemia (>7.0 mg/dL), chronic alcohol abuse, current or previous (6 months) recreational drug use, undergoing hemodialysis or any primary renal pathology, nephrotic syndrome or protein-losing syndrome, chronic obstructive pulmonary disease, current or previous diagnosis of malignant neoplasm, rheumatoid arthritis, and current use of dapsone, ribavirin, antiretroviral treatment, opioids, vitamins, and fluconazole which were not considered for the study. Approval was obtained from the Institutional Review Board and the Ethics Committee of our University.

### 2.2. Study Protocol

Patients were recruited at diagnosis of diabetic ketoacidosis or hypertriglyceridemia-induced pancreatitis and included in the study before starting any treatment for DKA or HTG-IP. Individuals underwent a comprehensive medical history including family and past medical history with special emphasis regarding conditions that may modify the HbA1c assay. In the case of diabetic ketoacidosis, arterial blood gas results were included in the medical record and the diagnosis was made according to the ADA criteria [16]. For hypertriglyceridemia-induced pancreatitis, amylase/lipase values were documented and diagnosed following the ACG recommendations [14].

Subsequently, an intravenous catheter (Vacutainer® 18G) was placed in a forearm vein and baseline blood samples (before diabetic ketoacidosis or hypertriglyceridemia-induced pancreatitis treatment) were drawn for the measurement of HbA1c, triglycerides, glucose, and hemoglobin. Following the same protocol, laboratory tests were repeated, in all cases, 24 and 48 hours after admission. The initial triglyceride sample was immediately processed to determine if it met the triglyceride inclusion criteria cutoff (≥150 mg/dL); the remaining samples were frozen at a temperature of −30°C (REVCO, Thermo Scientific, USA) in order to run all samples simultaneously and eliminate interassay error.

Patients with an index-hospital admission diagnosis of diabetic ketoacidosis and hypertriglyceridemia-induced pancreatitis were included in the study since both illnesses are characterized by an acute and transient elevation of triglycerides, which can be rapidly lowered within 24 to 72 hours with standard of care which is very similar in both pathologies (i.e., IV fluids, IV insulin, and electrolyte reposition). This allowed us to have an *in vivo* model to study the effect of different triglyceride values regarding HbA1c. Since triglyceride reduction was expected in a very short period of time (1-2 days) and all other known factors that impaired HbA1c were controlled, the potential change in HbA1c would have to be explained solely by a change in triglyceride levels.

### 2.3. Measurements

HbA1c was measured using ion-exchange HPLC (D-10™ Hemoglobin Analyzer, Bio-Rad, Hercules, California, USA) approved by the NGSP; intra-assay and interassay coefficients of variation (CV) were 0.78% and 0.52%, respectively. The HbA1c blood samples were frozen at −30°C until all the samples were collected. Additionally, hemoglobin (CELL-DYN Ruby, Abbott Laboratories, Abbott Park, IL, USA) and triglyceride levels and plasma glycemia (Unicel DxC 800 Synchron, Beckman Coulter Inc., Brea, CA, USA) were measured; intra-assay CV were 1.2%, 1.83%, and 2.0%, respectively. In order to reduce analytical variation, all samples were analyzed simultaneously and in duplicate.

### 2.4. Statistical Analysis

The sample size was calculated with a mean comparison formula using a difference of 0.05% between days 0 and 2 of HbA1c with a 95% level of confidence and 80% of power, estimating a total sample of 44 patients. Normality was assessed with the Kolmogorov-Smirnov test for all continuous data. Central tendency measures were mean and standard deviation in parametric data; median and interquartile ranges (IQR) were used for nonparametric distributed data. Frequency tables and percentages were used for categorical data. A two-tailed Student's *t*-test and one-way ANOVA were used to assess the differences between study groups for parametric data. The Mann-Whitney *U* and Kruskal-Wallis tests were used for nonparametric data. Categorical variables were compared using Pearson's chi-squared test or Fisher's exact test for 2 × 2 tables. A bivariate Pearson correlation was made between mean differences on days 0 and 2, days 0 and 1, and days 1 and 2 of HbA1c and triglycerides to assess for interference. A *p* value ≤0.05 was considered statistically significant. The statistical analysis was performed using IBM SPSS Statistics 22.0 (IBM Corp., Armonk NY, 2013).

## 3. Results

### 3.1. Study Population

A total of 44 patients were enrolled. The overall patients' baseline characteristics are shown in Table 1. Mean age was 31.4 years (SD ± 10.26), and almost two-thirds were men (65.9%). Most patients had diabetes (77.3%), and 50% had T2DM. Two-thirds of the included patients had diabetic ketoacidosis as an index-hospital admission diagnosis (63.6%), and there were 16 hypertriglyceridemia-induced pancreatitis (36.4%). The highest triglyceride value at baseline was 6451 mg/dL. Median triglyceride at baseline (day 0) was 722.5 mg/dL (IQR 378 to 2986), and this was significantly higher when compared to day one, 282 mg/dL (IQR 130 to 971), and day two, 258 mg/dL (IQR 119 to 580), *p* < 0.0001 and *p* < 0.0001, respectively. The difference in triglycerides between days 1 and 2 was also significant (*p* = 0.0020) (Table 2). No participant was lost to follow-up.

### 3.2. Comparison of HbA1c

Mean HbA1c values were compared over time at different triglyceride levels. Statistical but no significant clinical difference between mean serum HbA1c between days 1 and 2 of 0.16% [1.7 mmol/mol] (*p* = 0.003) was observed. No difference was found between days zero, 12.7%±3.22 [115 mmol/mol ± 35.2], and one, 12.7 ± 3.18 [115 mmol/mol ± 34.8] (*p* = 1.0), or zero and two, 12.6 ± 3.18 [114 mmol/mol ± 34.8] (*p* = 1.0).

**Figure 1:**
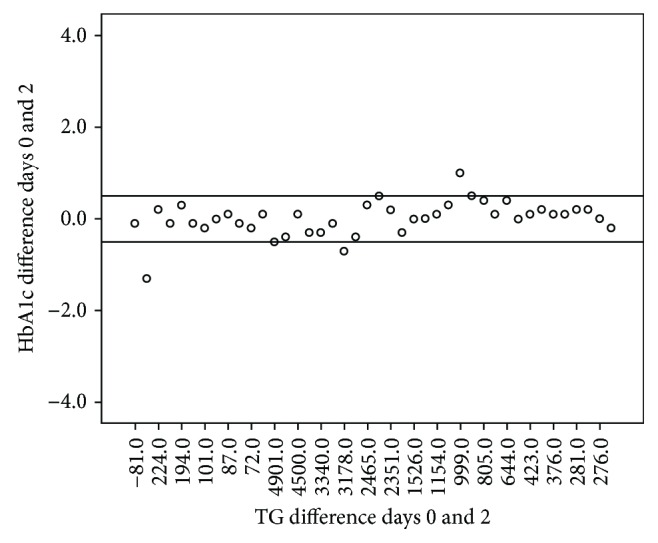
Differences between days 0 and 2 of HbA1c and triglycerides. TG: triglycerides. *N* = 43. Spearman nonparametric correlation (Spearman correlation coefficient = 0.265, *p* = 0.015).

Correlations between differences in HbA1c and triglyceride values were sought. From days zero to two, the mean difference in HbA1c when compared to the mean difference in triglycerides was found to be statistically significant; however, it was not clinically relevant (*p* = 0.256, *p* = 0.015) (Figure 1). We did not find any other significant correlation between mean differences in these variables from days zero to one or days zero to two.

### 3.3. Comparison between Diabetic Ketoacidosis and Hypertriglyceridemia-Induced Pancreatitis

A sensitive analysis was made between the two different index-hospital admission diagnoses (Table 3). Patients with pancreatitis had higher mean triglyceride values compared to diabetic ketoacidosis patients on days 0, 1, and 2 who had a mean difference of 2782 ± 505 mg/dL (*p* < 0.0001), 1484 ± 427 mg/dL (*p* < 0.0001), and 610 ± 172 mg/dL (*p* < 0.0001), respectively. Patients with pancreatitis were found to have a statistically significant difference between days 1 and 2 with a mean difference of 0.244%±0.08 [2.7 mmol/mol ± 0.9] (*p* = 0.024) with no clinically significant difference in HbA1c across the study period. We did not find differences in HbA1c (*p* = 0.135) among the studied days in patients with diabetic ketoacidosis.

### 3.4. Comparison between Subgroups of Triglycerides

We additionally performed a subanalysis based on baseline serum triglyceride values (group 1: <500 mg/dL (36.4%), group 2: 500-1000 mg/dL (20.5%), and group 3: >1000 mg/dL (43.2%)). A greater proportion of patients had triglycerides higher than 1000 mg/dL. There was a significant decrease in triglycerides between days 0, 1, and 2 within each group (*p* = 0.0001, *p* = 0.004, and *p* = 0.001, respectively), yet there was no difference in HbA1c within groups 1 and 2 (*p* = 0.56 and *p* = 0.21, respectively). However, there was a significant difference in group 3 between days 1 and 2 (mean difference, 0.24%±0.7) [2.6 mmol/mol ± 7.7] (*p* = 0.009) with no clinical significance (Table 4).

## 4. Discussion

### 4.1. Our Findings

In this prospective controlled study, we found that although triglycerides decreased substantially and there was a statistical difference in HbA1c (12.7% [115 mmol/mol] vs. 12.6% [114 mmol/mol], *p* = 0.004), this was far from having any clinical implications and should be considered normal due to the assays own expected intra-inter-assay variation. Likewise, while different index-hospital admission diagnoses (diabetic ketoacidosis or hypertriglyceridemia-induced pancreatitis) had a significant statistical difference for hypertriglyceridemia-induced pancreatitis (HbA1c, 9.8% [94 mmol/mol] vs. 9.7% [83 mmol/mol]), the clinical relevance of this remains not important to clinical practice. Finally, no difference was observed between HbA1c and higher triglyceride levels at baseline. To our knowledge, this is the first *in vivo* and prospective study evaluating the effect of hypertriglyceridemia on the HbA1c assay result.

### 4.2. Comparisons with Previous Studies

In some studies, it has been shown that triglycerides interfere heterogeneously in the HbA1c assay. Falko et al. [8] published a case report of spurious elevations in HbA1c in a woman with type 2 diabetes and hypertriglyceridemia (23,000 mg/dL). Since HbA1c did not properly correlate with glycemic control (HbA1*c* = 28.5%, fasting plasma glucose = 400 mg/dL), they further investigated these findings by washing out the patient's samples with saline solution to determine if triglycerides were interfering with the HbA1c determination. They concluded that when triglycerides were above 1750 mg/dL, HbA1c was falsely raised. Although these findings are interesting, due to the study design per se (case report), there was a decrease in the confidence of their results. Furthermore, the method used for HbA1c determination differs from HPLC (chromatographic method; Quik-Sep, Fast Hemoglobin Test Systems, Isolab, Akron, Ohio).

On the other hand, Garrib et al. [9] reported a case of falsely low glycated hemoglobin using an ion-exchange chromatographic method (Abbot Diagnostics, Maidenhead, Berkshire, UK). They found an HbA1c of 4.8% in a diabetic patient with blood glucose monitoring of 200-325 mg/dL and triglycerides of 2388 mg/dL. Similar to the previous study, they washed out the samples with saline solution and retested the assay, reporting a more accurate HbA1c for the patients' glycemic control (HbA1c, 12.2%). In addition, they reviewed the HbA1c and triglycerides of 98 diabetic patients finding a significant increase in HbA1c in the washed samples when triglycerides were >1320 mg/dL (*p* = 0.0001). Moreover, a significant positive correlation between triglycerides and the difference of HbA1c washed and unwashed was found (*R* = 0.51, *p* = <0.001) as well as between the HbA1c differences (*R* = 0.65, *p* = <0.005). They additionally compared their results with two other different methods (the Delta immunoturbidimetric method and HPLC). Interestingly, they found no significant correlation using these methodologies (HPLC: *R* = 0.018, *p* = <0.66; Delta: *R* = 0.007, *p* = 0.79). Although findings from previous studies demonstrate triglycerides as an interference variable in HbA1c, none of them were performed under *in vivo* conditions nor was HPLC used as their main method. In our study, a statistically significant difference in HbA1c was found; however, it was not clinically significant. We infer that the change in HbA1c was not due to triglycerides itself. Some other confounding variables such as the normal intraindividual CV, which some studies had reported from 0.7 to 0.9%, might be the cause of the minor HbA1c differences found [18, 19].

### 4.3. Implications for Clinical Practice

Hypertriglyceridemia is common in patients with type 2 diabetes (20-60%) [11]. Until now, clinicians and patients face the common clinical scenario of uncertainty when interpreting HbA1c levels in the light of high triglyceride levels. As recently reported, data from nonclinical and case report studies has led to misleading statements based on low-quality evidence about the interference produced by hypertriglyceridemia over HbA1c results [20]. Our data strongly suggest that there is no HbA1c impairment by triglycerides. Thus, being HbA1c the main test used to assess glycemic control and a diabetes diagnostic method [21], the results of our study provide useful and relevant data that should be applied in daily clinical practice. Also, the prevalence of lipemia (triglycerides ≥1000 mg/dL) is found in around 0.5–2.5% of the population [22], and our result strongly suggests that clinicians should not consider high levels of triglycerides as a relevant analytical interference factor when interpreting HbA1c results.

### 4.4. Strengths and Limitations

Strict protocol and finding an *in vivo* model in which high triglyceride levels in diabetic ketoacidosis or hypertriglyceridemia-induced pancreatitis can be rapidly reverted within 24-72 hours with standard treatment is a strength in our study. This real-life model allowed us to satisfactorily evaluate if the rapid decrease in triglyceride levels impaired the interpretation of the HbA1c assay, and to our knowledge, no prior *in vivo* study of triglycerides as an interference variable in the HbA1c assay has been reported. In addition, none of the patients was lost to follow-up. Several limitations reduce the confidence in our results, being the most important not having a nonill control group; however, each of the study subjects was their own control, and in a nonill control group, decreasing triglycerides that fast would have been very difficult.

## 5. Conclusion

Triglycerides do not impair, in a clinically significant manner, the performance of HbA1c measurement. Acknowledging this has important clinical implications in day-to-day clinical practice as many patients with uncontrolled hyperglycemia will have concomitant hypertriglyceridemia. With this data, patients and clinicians can now have more confidence that hypertriglyceridemia is not a factor to take into account when interpreting HbA1c results.

## Figures and Tables

**Table 1:** Demographic characteristics.

Characteristics	Mean (percent)
	*N* = 44
Age in years—mean (SD)	31.43 (10.2)
Male sex—no./total no. (%)	28 (65.9)
Diabetes—no. (%)	34 (77.3)
Type 1	12 (27.2)
Type 2	22 (50)
Triglyceride-induced pancreatitis—no. (%)	16 (36.4)
Diabetic ketoacidosis—no. (%)	28 (63.6)
Mean HbA1c—mean (SD) (%, mmol/mol)	
Day 0	12.7 (3.22) [115 (35.2)]
Day 1	12.7 (3.18) [115 (34.8)]
Day 2	12.6 (3.18) [114 (34.8)]
Median TG—median (IQR)	
Day 0	722.5 (378-2986)
Day 1	282 (130-971)
Day 2	258 (119-580)

**Table 2:** Mean HbA1c changes.

Day	0	1	2	*p* value
HbA1c (%, mmol/mol)	12.7 ± 3.2 [115 ± 35.2]	12.7 ± 3.1 [115 ± 34.8]	12.6 ± 3.1 [114 ± 34.8]	0.004
Days 0-1	-0.06 [-0.6]	—	—	1.00
Days 1-2	0.16 [1.7]	—	—	0.003
Days 2-0	0.09 [1]	—	—	1.00

ANOVA of repeated measure and post hoc analysis using Bonferroni correction for multiple comparisons.

**Table 3:** Mean HbA1c and TG among groups (DKA and HTG-IP).

Hba1c (%, mmol/mol), mean (±SD)	Day 0	Day 1	Day 2	*p* value	Triglycerides, median (IQR)	Day 0	Day 1	Day 2	*p* value
Pancreatitis	9.8 (3.1)	10 (3.1)	9.7 (3.1)	0.028	—	3566 (1874-4912)	1018 (652-2438)	620 (495-793)	0.0002
	84 (33.9)	86 (33.9)	83 (33.9)						

Diabetic ketoacidosis	14.3 (1.7)	14.3 (1.8)	14.2 (1.7)	0.135	—	493 (313-783)	169 (97-258)	147 (97-248)	<0.0001
	133 (18.6)	133 (19.7)	132 (18.6)						

**Table 4:** Comparison between triglyceride subgroups.

	^∗^TG <500 *N* (16)	*p* value	TG 500-1000 *N* (9)	*p* value	TG >1000 *N* (19)	*p* value
HbA1c (%, mmol/mol)						
Day 0	13.4 ± 1.9 [123 ± 20.8]	0.56	14.2 ± 2.1 [132 ± 23]	0.21	11.3 ± 3.9 [100 ± 42.6]	0.007
Day 1	13.4 ± 1.8 [123 ± 19.7]	—	14.4 ± 1.9 [134 ± 20.8]	—	11.3 ± 3.9 [100 ± 42.6]	—
Day 2	13.3 ± 1.7 [122 ± 18.6]	—	14.3 ± 1.8 [133 ± 19.7]	—	11.1 ± 3.9 [98 ± 42.6]	—
TG						
Day 0	322.5 (188.5)	0.0001	695.0 (237.0)	0.004	3334.0 (3281.0)	0.0001
Day 1	182.5 (111.2)	—	186.0 (279.5)	—	984.0 (1263)	—
Day 2	163.5 (112.3)	—	172.0 (215.5)	—	619 (419)	—

^∗^TG: triglycerides.

## Data Availability

The data used to support the findings of this study are available from the corresponding author upon request.
